# Prevention of Lead Colic

**Published:** 1843-04

**Authors:** 


*Prevention of Lead Colic.*—Mr. Benson, the managing director at the British white lead works, Birmingham, says that the use of what he calls sulphuric beer by the workmen is an effectual preventive of the colic arising from the effects of the lead, and which, previously to the employment of the sulphuric beer, was exceedingly prevalent among the men. He was induced to try it from a statement made some time since, that sulphuric lemonade had been successfully used at a white-lead manufactory in France. Its action must be effected by the chemical transformation of the poisonous carbonate into the innocuous sulphate of lead. The formula for the preparation of the beer is as follows;—Take of treacle, fifteen pounds; bruised ginger, half a pound; water, twelve gallons yeast, one quart; bicarbonate of soda, one ounce and a half sulphuric acid, one ounce and a half by weight. Boil the ginger in two gallons of water; add the treacle and the remainder of the water hot. When nearly cold, transfer it to a cask, add the yeast, to cause fermentation. When this has nearly ceased, add the sulphuric acid, previously diluted with eight times its quantity of water, and then the bicarbonate of soda, dissolved in a quart of water. Close up the cask, and in three or four days the beer will be fit for use. As acetous fermentation speedily takes place, particularly in hot weather, new supplies should be prepared as required. The object in adding the bicarbonate of soda is to give a pleasant briskness to the beverage. The sulphuric acid remains greatly in excess.—*Lancet*, Dec. 17, 1842.

